# Disclosures Respecting Spirit-Rapping

**Published:** 1854-10

**Authors:** 


It will be remembered by the readers of the *Western Journal of Medicine*, that Prof. Flint and some other medical gentlemen of Buffalo detected the Misses Fox, a few years since, in the act of producing *Spirit-rapping* with their knees. A similar case is given in the following extract from the *London Lancet*, for September:—

Disclosures respecting Spirit-Rapping.—At the meeting of the Academy of Sciences of Paris, on the 12th of June last, M. Rayer called the attention of the members to some experiments of Dr. Schiff, of Frankfort, touching spirit-rapping. This physician had under his observation a young girl, with whom certain noises took place, attributed to the rapping of spirits. Dr. Schiff, by paying much attention to the case, found out that the sound issued from the girl herself, and not from any other source. The noise is, in fact, produced by the slipping of the tendon of the peronæus longus from the sheath in which it glides in passing behind the outer ankle. Dr. Schiff succeeded in repeating upon himself the same phenomena presented by the girl, which was supposed to depend upon a rapping spirit. When the sheath of the tendon is weak and lax the noise is very easily made, and may be produced without moving the foot in the least; but when the finger is placed behind the outer ankle just when the sound is being made, the alternate and repeated displacement is felt without difficulty, the action being accompanied by a rapid up and down movement of the same tendon. The Secretary of the Academy requested Dr. Schiff, who was present at the meeting, to repeat the experiment before the members. The rapping was accordingly produced, and heard at the distance of several yards, though silence was far from being complete. The feet were quite open to view, but no movement could be perceived.

